# Gestational Trophoblastic Neoplasm Favouring Epithelioid Trophoblastic Tumour

**DOI:** 10.7759/cureus.115361

**Published:** 2026-08-28

**Authors:** Diogo Carvalho, Delfim Doutel, Teresa Margarida Cunha

**Affiliations:** 1 Radiology, Instituto Português de Oncologia de Lisboa Francisco Gentil, Lisbon, PRT; 2 Pathology, Instituto Português de Oncologia de Lisboa Francisco Gentil, Lisbon, PRT

**Keywords:** case report, epithelioid trophoblastic tumour, gestational trophoblastic disease (gtd), magnetic resonance imaging, uterine tumour

## Abstract

Epithelioid trophoblastic tumour (ETT) is a rare form of gestational trophoblastic disease (GTD). We report a case of a 28-year-old woman presenting with abnormal uterine bleeding and mildly elevated serum beta human chorionic gonadotropin (β-hCG) levels. Imaging revealed a heterogeneous uterine mass with lymphadenopathy and pulmonary metastases. Core biopsy histology suggested ETT, but definitive subclassification was limited by small sample size and an ambiguous immunohistochemical profile, which is itself a recognised source of diagnostic uncertainty in this entity. The patient received neoadjuvant platinum-based chemotherapy, followed by total hysterectomy with bilateral salpingo-oophorectomy and lymphadenectomy. At 10 months postoperatively, the patient remains disease-free with normalisation of β-hCG levels. This case illustrates the diagnostic challenges inherent to GTD, particularly the overlap between ETT, placental site trophoblastic tumour (PSTT) and choriocarcinoma; emphasises the importance of integrating multimodal imaging with histopathology within a multidisciplinary team; and demonstrates that a combined approach of chemotherapy and surgical resection can lead to favourable outcomes in this rare entity.

## Introduction

Gestational trophoblastic disease (GTD) encompasses several malignant entities, including invasive mole, choriocarcinoma, placental site trophoblastic tumour (PSTT), and epithelioid trophoblastic tumour (ETT). ETT is a rare form of GTD arising from intermediate trophoblasts at the placental implantation site, developing from chorionic-type intermediate trophoblasts. It accounts for approximately 1-2% of all gestational trophoblastic neoplasms [1,2], making it the rarest member of the GTD spectrum. ETT typically affects women of reproductive age and often presents months to years after pregnancy, most commonly following non-molar gestations. ETT is generally slow-growing, with a tendency for local uterine invasion and preferential lymphatic spread, while haematogenous dissemination is less frequent. Although ETT shares overlapping morphologic and clinical features with other forms of GTD, differences in growth pattern and histopathologic characteristics aid in their distinction [3-5].

## Case presentation

A 28-year-old woman (G2P2) presented with a one-month history of abnormal uterine bleeding, three years after an uncomplicated vaginal delivery. Serum beta human chorionic gonadotropin (β-hCG) was persistently elevated, rising from 133 mIU/mL at initial presentation to 136 mIU/mL one week later and 176 mIU/mL approximately one month later, before peaking at 815 mIU/mL (reference range: <5 mIU/mL) prior to the initiation of chemotherapy. Transvaginal ultrasound revealed an indeterminate posterior uterine mass without intrauterine gestation or adnexal abnormality.

Given the predominantly myometrial and subserosal location of the mass, its posterior and non-intracavitary extension, and the absence of an intrauterine gestational sac on ultrasound, a percutaneous image-guided core needle biopsy was chosen over dilation and curettage or hysteroscopic biopsy. Endometrial sampling techniques were considered unlikely to reach the lesion given its predominantly extraluminal growth, whereas core biopsy allowed direct, targeted sampling of the myometrial component with an acceptable safety profile.

**Figure 1:**
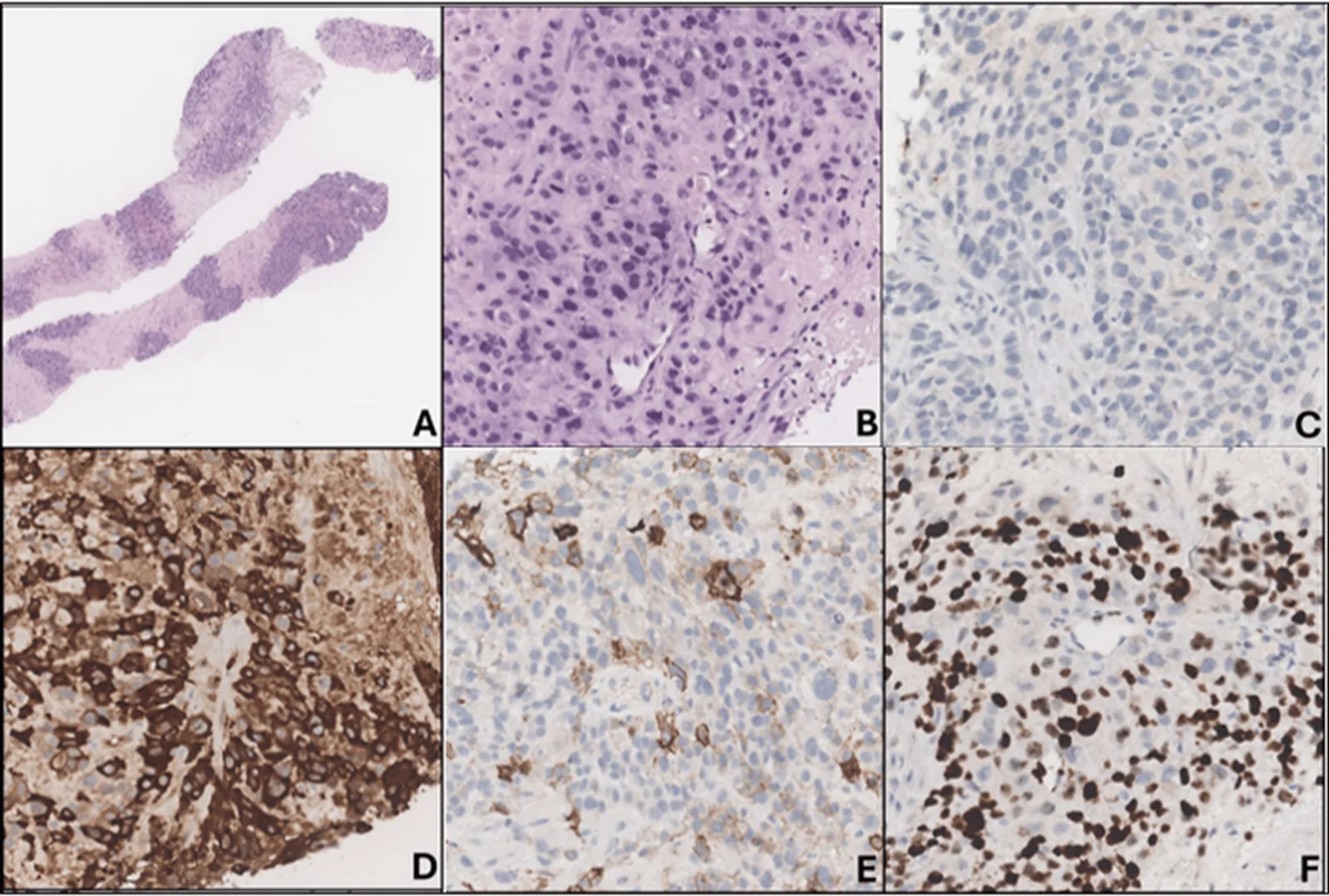
Histopathologic features of the uterine core biopsy specimen. Malignant gestational trophoblastic neoplasm with characteristics suggestive of epithelioid trophoblastic tumor. (A) Low-power view (H&E) showing striking geographic necrosis, with pale, band-like necrotic areas alternating with darker, viable tumor nests. (B) Medium-power view (H&E) showing preservation of tumor cell nests around small vessels. The neoplastic cells are relatively uniform and epithelioid with abundant cytoplasm and nuclear atypia. There are 13 mitoses/10 high-power fields. (C) SALL4 immunohistochemistry is negative, with only the blue hematoxylin counterstain visible and no brown staining within the tumor nests. (D, E) Human placental lactogen (hPL) and placental-like alkaline phosphatase (PLAP) immunohistochemistry, respectively, show heterogeneous expression, with patchy brown cytoplasmic staining in some tumor cells and no staining in others. (F) Ki-67 shows brown nuclear staining in more than 50% of the neoplastic cells.

Core biopsy showed a malignant gestational trophoblastic neoplasm suggestive of ETT, though definitive classification was limited by an ambiguous immunophenotype and small sample size (Figure 1).

**Figure 2:**
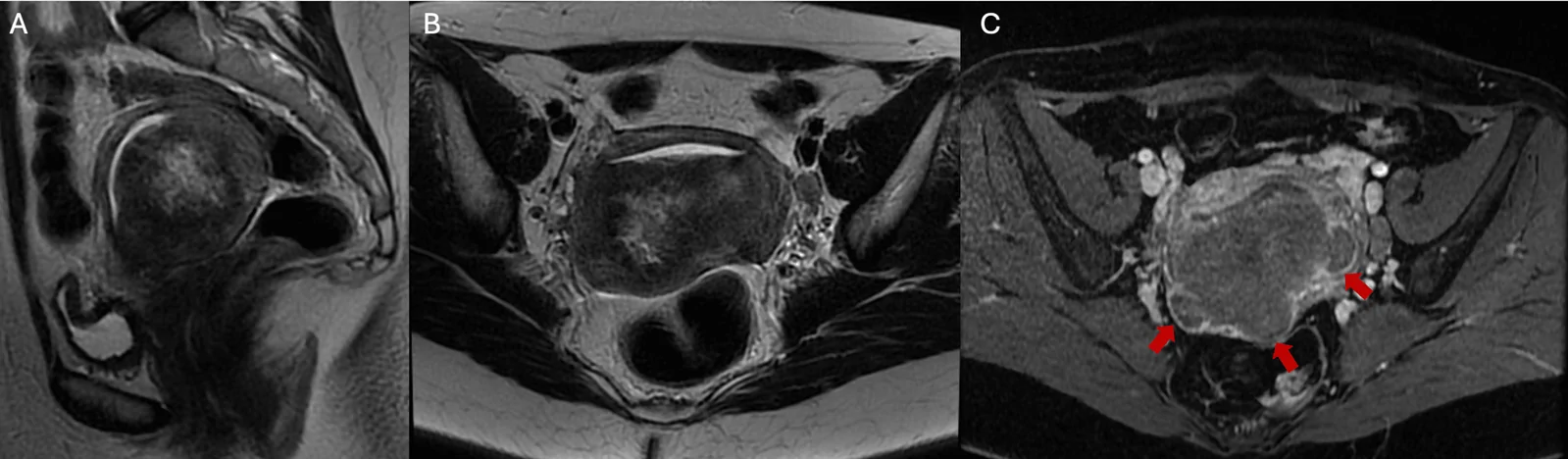
MRI features of the uterine tumour. Sagittal T2-weighted (A) and axial T2-weighted (B) images of the pelvis demonstrate a heterogeneous tumour centred in the posterior uterine body. The axial T1 fat-saturated post-gadolinium image (C) shows the tumour to be hypointense relative to the surrounding myometrium, with invasion of the outer myometrium at three distinct sites (red arrows), without disruption of the serosa.

**Figure 3:**
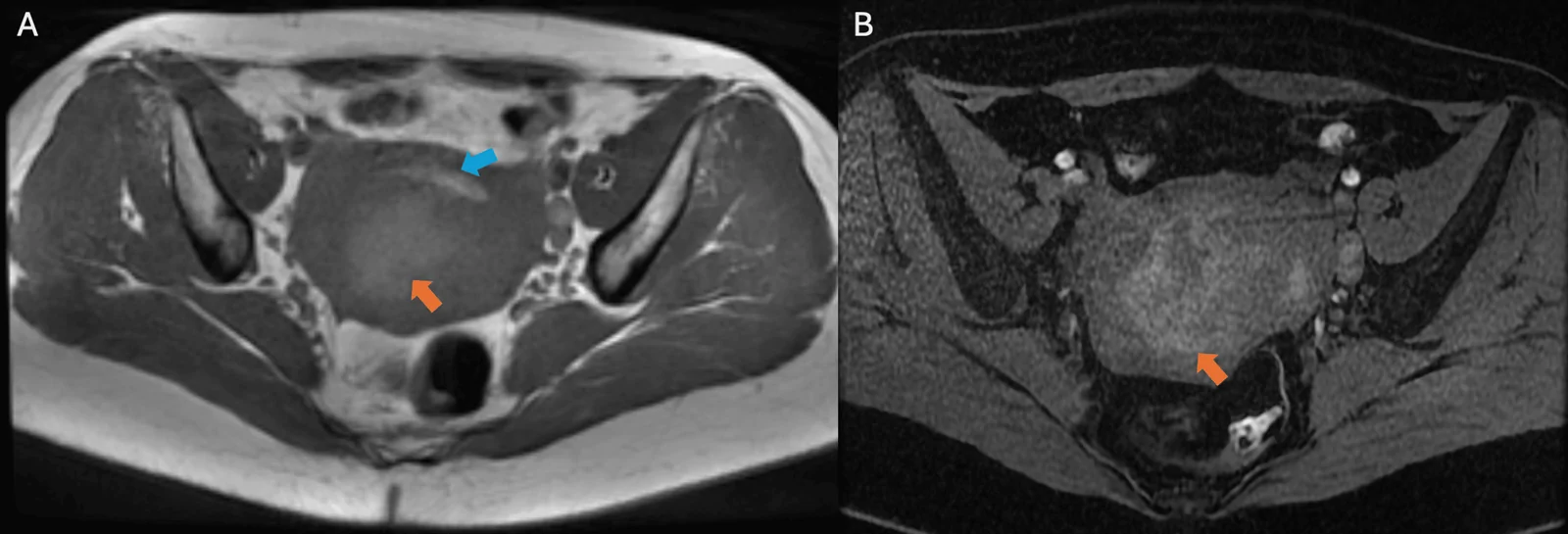
MRI features of the uterine tumour. Axial T1-weighted and T1 fat-saturated images (A, B) show a small hematometra (blue arrow, A) and intralesional haemorrhage (orange arrows).

**Figure 4:**
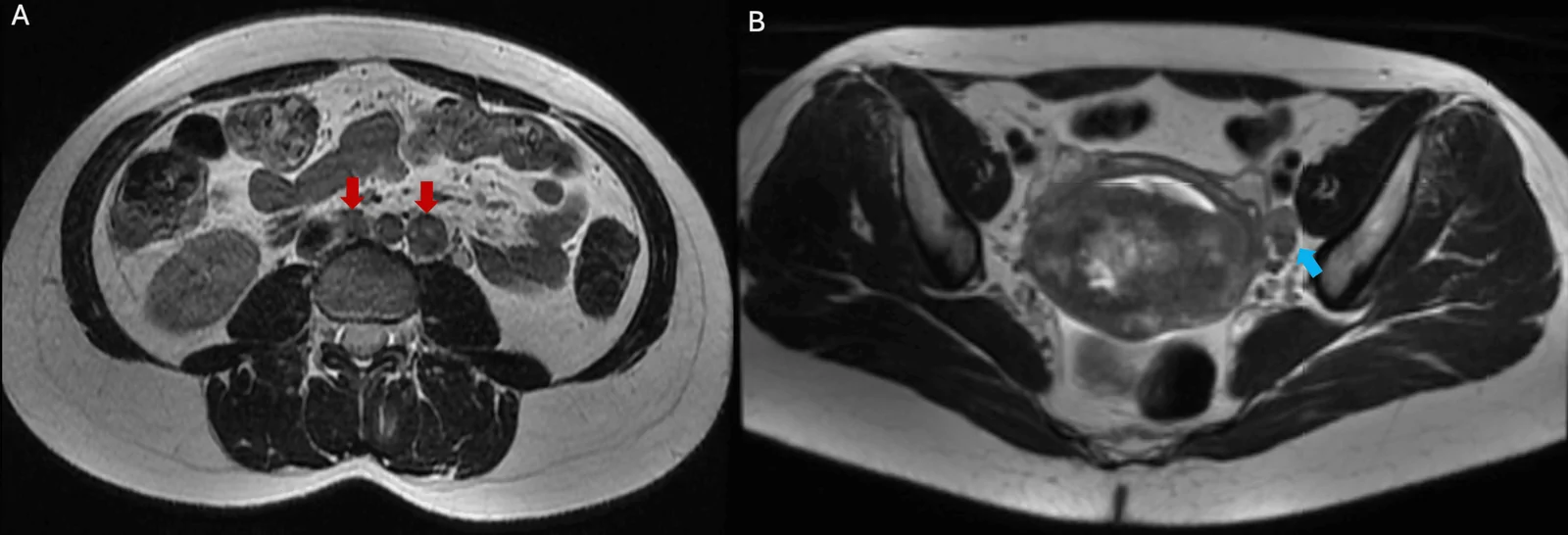
MRI demonstrating retroperitoneal and pelvic lymphadenopathy. T2-weighted axial images demonstrate enlarged lymph nodes in the interaortocaval and left para-aortic regions (red arrows in A), as well as within the posterior group of the left external iliac chain (blue arrow in B).

**Figure 5:**
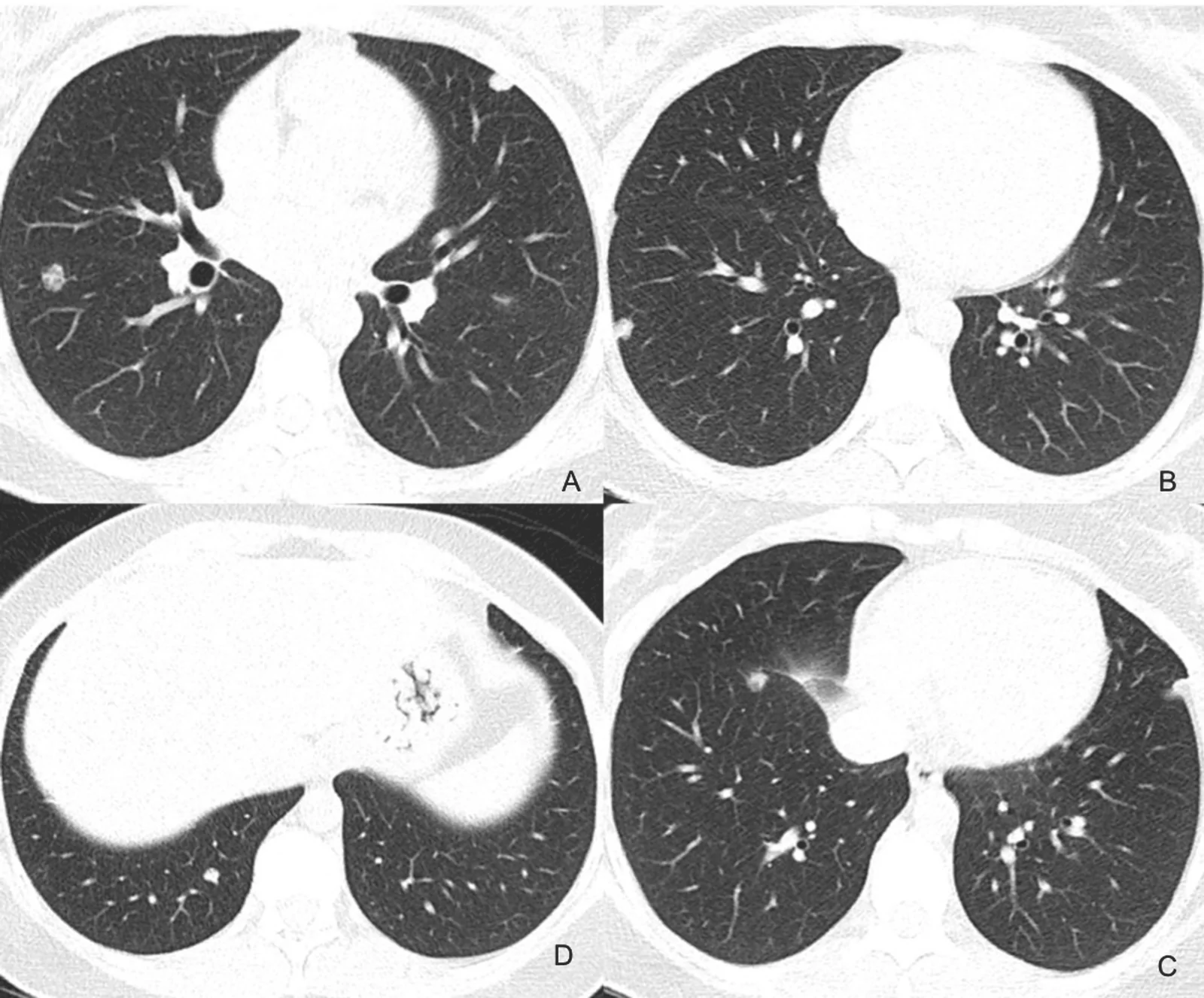
CT of the thorax demonstrating multiple lung metastases. Axial CT images of the thorax at four different levels, arranged clockwise from the most superior level (A) to the most inferior level (D), demonstrate multiple bilateral pulmonary nodules consistent with metastases.

Magnetic resonance imaging (MRI) demonstrated a solid, heterogeneous mass in the posterior uterus infiltrating the outer myometrium and abutting the serosa at multiple sites, without definite evidence of serosal breach, measuring approximately 76 x 66 x 47 mm in transverse, longitudinal, and anteroposterior axes, respectively (Figure 2). Intralesional haemorrhage and a small haematometra were also present (Figure 3). Enlarged left para-aortic, interaortocaval, and left external iliac lymph nodes were noted, with short-axis diameters ranging between 11 and 15 mm (Figure 4). Chest computed tomography (CT) revealed multiple bilateral lung nodules suspicious for metastases, the largest measuring approximately 9 mm (Figure 5).

**Figure 6:**
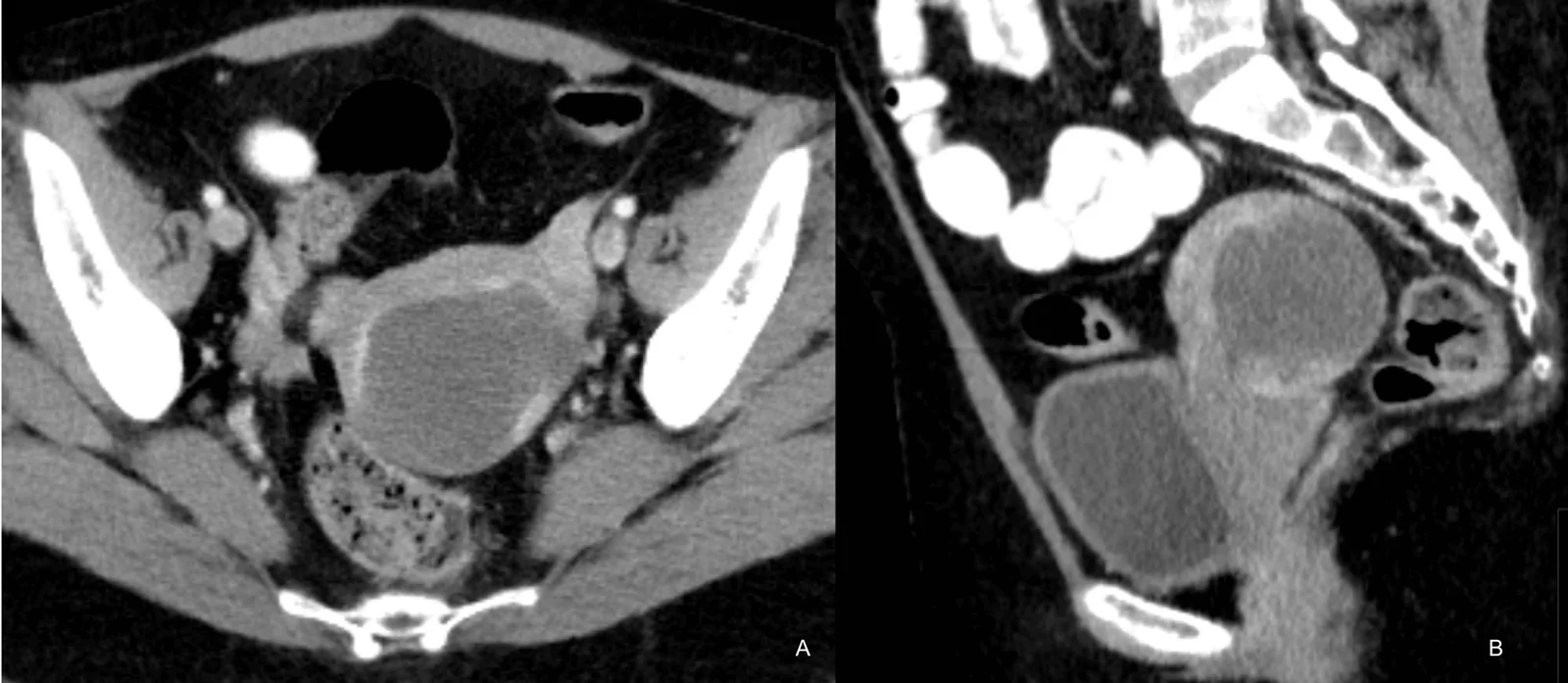
CT of the pelvis demonstrating persistence of the uterine tumour following chemotherapy. Axial (A) and sagittal reformatted (B) CT images of the pelvis both demonstrate persistence of the uterine tumour following chemotherapy.

The patient received six cycles of platinum-based chemotherapy. Follow-up CT showed regression of nodal and pulmonary disease but persistent uterine involvement, with the tumour measuring approximately 60 x 49 x 52 mm in transverse, longitudinal, and anteroposterior axes, respectively (Figure 6), prompting total hysterectomy with bilateral salpingo-oophorectomy and lymphadenectomy. The surgical specimen showed extensive necrosis with only scant residual trophoblastic cells, precluding definitive classification.

Follow-up MRI at six weeks showed no residual disease, and thoracic CT at four months demonstrated near-complete resolution of the pulmonary nodules. Serum β-hCG remained normal at 2.1 mIU/mL ten months post-surgery.

## Discussion

GTD encompasses a heterogeneous group of neoplasms, such as ETT, PSTT, and choriocarcinoma, with overlapping clinical, radiologic, and pathologic features, complicating subtype classification in atypical presentations, as in this case. Distinguishing between them relies on the combination of morphology, immunohistochemistry, and serum β-hCG kinetics.

Diagnostic criteria for ETT and distinction from PSTT and choriocarcinoma

Morphologically, ETT is composed of relatively monomorphic, mononuclear intermediate trophoblasts with abundant eosinophilic-to-clear cytoplasm, arranged in nests and cords with a well-circumscribed, nodular growth pattern; a characteristic feature is geographic (map-like) necrosis, with preservation of viable tumour cell nests around small vessels and an eosinophilic, hyaline-like extracellular matrix reminiscent of keratin, which frequently leads to confusion with squamous cell carcinoma [1,5,6]. Immunohistochemically, ETT is typically positive for p63 throughout, with only minimal or no staining for β-hCG and human placental lactogen (hPL). The Ki-67 proliferation index is generally in the range of 10-25% [1,3,7].

PSTT typically shows a diffuse, sheet-like, infiltrative growth pattern rather than the nested, well-circumscribed architecture of ETT, and its tumour cells are strongly and diffusely positive for hPL, in contrast to the focal or absent hPL expression seen in ETT [4,7]. Importantly, p63, which is typically expressed strongly and diffusely throughout the tumour cells in classic ETT [8], is characteristically negative in PSTT, making it a useful marker for distinguishing the two entities [8,9]. PSTT also more frequently arises in the uterine corpus rather than the lower uterine segment or cervix. Ki-67 proliferation indices show substantial overlap between PSTT and ETT and are therefore of limited value for their distinction [8].

Distinguishing ETT from choriocarcinoma relies on both morphology and serum β-hCG kinetics. The morphological appearances of a choriocarcinoma show the three cell lineages (chorionic-type trophoblasts, implantation-type trophoblasts, and syncytiotrophoblasts) in varying proportions, and variable degrees of necrosis and haemorrhage, sometimes seen as blood lakes or pseudovascular channels. Characteristically, they show strong β-hCG positivity, whereas ETT shows focal or no reactivity. SALL4 is a potentially further useful discriminator, being negative in ETT and PSTT and with more than 10% staining overall in choriocarcinomas [9]. Clinically, serum β-hCG in choriocarcinoma is usually markedly elevated (often >10,000-100,000 mIU/mL), whereas in ETT it is typically modestly elevated, below 2,500 mIU/mL in most series [7,10], consistent with the peak value of 815 mIU/mL observed in our patient.

Imaging can also support, though not confirm, this distinction. In our patient, the MRI appearance, a heterogeneous myometrial mass with relatively modest post-contrast enhancement and areas of haemorrhage and necrosis, but without the marked hypervascularity and arteriovenous shunting typically described in choriocarcinoma, was more in keeping with ETT or PSTT than with choriocarcinoma [4,11], consistent with previous descriptions of ETT on MRI as a well-circumscribed mass with heterogeneous, often modest, gadolinium enhancement [4,11]. This appearance is not specific, however, and reliable differentiation between ETT, PSTT, and choriocarcinoma cannot be made on imaging alone [4,12]; reported appearances of ETT itself range from predominantly cystic masses with mural nodules to solid heterogeneous lesions as in this case [2,11-13], and the tumour can mimic other gestational and non-gestational malignancies. Table 1 summarizes the comparative clinical, radiologic, and immunohistochemical features of ETT, PSTT, and choriocarcinoma.

**Table 1:** Comparative clinical, radiologic, and immunohistochemical features of ETT, PSTT, and choriocarcinoma. Sources: [1,3,4,7,9,10,14,15]

Feature	Epithelioid trophoblastic tumour (ETT)	Placental site trophoblastic tumour (PSTT)	Choriocarcinoma
Cell of origin	Chorionic-type intermediate trophoblast	Implantation-site intermediate trophoblast	Syncytiotrophoblast, cytotrophoblast and intermediate trophoblast
Growth pattern/microscopic characteristics	Nested, well-circumscribed, nodular; large areas of geographic necrosis are usually visible	Diffuse, sheet-like, infiltrative	Infiltrative and destructive; background necrosis and haemorrhage
Typical location	Lower uterine segment/cervix, or uterine body	Uterine corpus	Uterine corpus; may follow any pregnancy type
Serum β-hCG	Modest elevation, usually <2,500 mIU/mL	Wide range, ~5–26,000 mIU/mL; generally lower than choriocarcinoma	Markedly elevated, often >10,000-100,000 mIU/mL
Key positive IHC markers	p63 (diffuse)	hPL (diffuse), CD146 (Mel-CAM)	β-hCG (diffuse)
Key negative/low markers	Β-hCG, hPL, SALL4	Β-hCG, p63, SALL4	
Ki-67 index	10–25%	10–30%	>90%
Primary route of spread	Lymphatic (before haematogenous)	Lymphatic	Haematogenous
Chemosensitivity	Relatively chemoresistant	Relatively chemoresistant	Highly chemosensitive
Primary treatment	Surgical resection ± chemotherapy	Hysterectomy ± chemotherapy	Chemotherapy (surgery reserved for residual/complications)

Interpretation of the immunohistochemical profile in this case

In our patient, the tumour cells expressed AE1/AE3, GATA3, and inhibin, with heterogeneous placental-like alkaline phosphatase (PLAP) and hPL expression, and were negative for SALL4, β-hCG, and p63. GATA3, AE1/AE3, and inhibin positivity confirm a trophoblastic lineage but, being expressed across all trophoblast subtypes, do not by themselves distinguish between ETT, PSTT, and choriocarcinoma [8]. The focal/heterogeneous, rather than diffuse, hPL expression argues against PSTT; the p63 negativity, meanwhile, argues against ETT and is more typical of PSTT. SALL4 negativity is expected in ETT and PSTT but does not rule out choriocarcinoma [14,15]. β-hCG negativity likewise points away from choriocarcinoma, although not conclusively: choriocarcinoma comprises a variable admixture of chorionic-type, implantation-type, and syncytiotrophoblastic elements, and since only the syncytiotrophoblastic component produces β-hCG, a sample in which it is scant may still appear negative [8]. The Ki-67 proliferation index in the core biopsy, at over 50%, is a further point of ambiguity: this is higher than typically reported for either ETT or PSTT, though still below the very high levels (>90%) characteristic of choriocarcinoma [8]. This unexpected combination of morphology favoring ETT but an ambiguous immunohistochemical profile is precisely what limited definitive subclassification on the small core biopsy in this case. On the resection specimen, extensive treatment-related necrosis with only scant residual trophoblastic cells similarly precluded resolution of this ambiguity, and in retrospect, a mixed or transitional ETT/PSTT phenotype, or a choriocarcinoma component undersampled on biopsy, cannot be entirely excluded.

Influence of diagnostic uncertainty on treatment planning

The decision to proceed with neoadjuvant platinum-based chemotherapy before surgery was driven principally by the presence of metastatic disease at presentation, for which combined chemotherapy and surgery is standard in GTN [3]; the inability to exclude choriocarcinoma on the initial biopsy, given its markedly better chemosensitivity than ETT or PSTT [4,7], further supported this sequencing. Had a confident preoperative diagnosis of pure ETT been available, the overall treatment sequence would likely have been similar, since surgery is recommended as the primary treatment for ETT confined to the uterus and combined surgery plus multi-agent chemotherapy is recommended for metastatic disease [3]; a confirmed diagnosis of choriocarcinoma, in contrast, might have supported chemotherapy alone as definitive treatment, without hysterectomy, underscoring why resolving this ambiguity, where feasible, is clinically meaningful.

Comparison with previously reported metastatic ETT

Metastatic disease is reported in approximately 25-43% of ETT cases at diagnosis, with the lung representing the most frequent extrauterine site, followed by the vagina, pelvic and retroperitoneal lymph nodes, liver, and brain [1,3,7]. A recent literature review compiling approximately 53 reported cases of uterine ETT with metastatic or extrauterine disease found considerable heterogeneity in outcome, with regimens such as EMA-CO (etoposide, methotrexate, actinomycin-D, cyclophosphamide, vincristine) and EMA-EP (etoposide, methotrexate, actinomycin-D/etoposide, cisplatin) most commonly used, and combined surgical resection with multi-agent chemotherapy generally yielding the most favourable outcomes, while chemotherapy alone was frequently insufficient to achieve durable remission [7]. In a multicentre series of 31 patients with ETT, the number of metastatic sites was the strongest independent predictor of overall and recurrence-free survival: patients with three or more metastatic sites had markedly worse survival (33.3% at five years) than those with fewer than three (100% at five years), and chemotherapy without surgery was independently associated with recurrence [3].

Our patient presented with pulmonary, para-aortic, interaortocaval, and iliac nodal disease in addition to the uterine mass, which, based on the above series, would predict a comparatively unfavourable prognosis. Despite this, she achieved a near-complete radiological response of the metastatic disease with platinum-based chemotherapy and remains disease-free with normal β-hCG at 10 months, an outcome that compares favourably with previously reported multi-site metastatic ETT [3,7]. This relatively favourable response may reflect a shorter interval from antecedent pregnancy (three years). An interval of ≥48 months since the antecedent pregnancy has been reported as an adverse prognostic factor in PSTT, and was similarly identified as a prognostic factor for ETT/PSTT in the 54-patient International Society for the Study of Trophoblastic Disease (ISSTD) database analysis by Frijstein et al. [16], as discussed by Liu et al., although this specific interval was not confirmed as an independent predictor in Liu et al.'s own multivariate analysis of 31 patients [3]. Our patient's three-year interval remains well below this reported threshold. The favourable response may otherwise reflect a possible choriocarcinoma or other more chemosensitive component not excluded by limited sampling, or simply the recognised heterogeneity of ETT behaviour; longer follow-up will be required to confirm durability of this response, consistent with the late-recurrence potential described for ETT and PSTT [3,4].

Multidisciplinary management and follow-up

The management of this case relied on close collaboration between radiology, pathology, gynaecologic oncology, and medical oncology, from initial imaging characterisation and biopsy planning through interpretation of an ambiguous immunophenotype, selection and sequencing of chemotherapy and surgery, and postoperative surveillance. Given the rarity of ETT and the lack of large randomised trials, this multidisciplinary discussion was instrumental in reaching a pragmatic management strategy in the face of diagnostic uncertainty, and is consistent with current recommendations for the management of GTD [3,17].

Given the potential for late recurrence, long-term surveillance incorporating serial β-hCG measurement and imaging is essential [5]. International guidance on GTD, most recently updated by Ngan et al. [18], supports structured post-treatment follow-up combining periodic β-hCG measurement with clinical and, where indicated, radiologic surveillance. A commonly cited surveillance schedule for patients with gestational trophoblastic neoplasia in remission consists of serum β-hCG determinations every two weeks for the first three months, followed by monthly measurements for at least 12 months; because the risk of recurrence beyond the first year of remission remains higher in high-risk disease such as ETT/PSTT, hCG monitoring is typically continued at six- to 12-month intervals thereafter [17,18]. Radiologic surveillance is less rigidly standardised than β-hCG monitoring: rather than following a fixed imaging calendar, most reported series obtain follow-up imaging near completion of treatment to document response, with some authors additionally recommending repeat imaging if serum β-hCG rises during surveillance, rather than at fixed time points [19]. The early post-treatment imaging performed in our patient (pelvic MRI at six weeks; chest CT at four months) is consistent with this pattern; notably, residual pulmonary lesions are reported in up to 19-30% of patients with normal β-hCG after chemotherapy for GTN and do not appear to confer an increased risk of relapse, which is reassuring when interpreting minor post-treatment imaging findings in this population [20]. This patient has been followed according to this framework, with early post-treatment imaging showing resolution of disease and normal β-hCG at 10 months.

## Conclusions

This case illustrates the diagnostic challenge of GTD with nonspecific imaging findings and limited biopsy material, and highlights the specific difficulty of distinguishing ETT from PSTT and choriocarcinoma when the immunohistochemical profile is ambiguous. The integrated clinical context, β-hCG profile and kinetics, imaging characteristics, and core biopsy histology most strongly favoured ETT within the GTD spectrum, though the ambiguous immunoprofile meant that PSTT and, to a lesser extent, choriocarcinoma could not be entirely excluded. Given the metastatic disease and the relative chemoresistance reported in ETT and select GTD subtypes, management was oriented towards a combination of chemotherapy and surgical resection with long-term surveillance, achieved through close multidisciplinary collaboration between radiology, pathology, and gynaecologic and medical oncology. This uncommon infiltrative presentation, with a comparatively favourable outcome despite multisite metastatic disease, broadens radiologic and clinical awareness of atypical GTD manifestations suggestive of ETT, and underscores the value of multidisciplinary evaluation in diagnostically challenging cases.

## References

[1] Kumar A, Diwan H, Sharma A, Kamboj M (2023). Epithelioid trophoblastic tumors: a diagnostic challenge. Int J Gynecol Cancer.

[2] Idegami D, Amano T, Torii H, Tsuji S, Urabe M, Murakami T (2024). Characteristics of epithelioid trophoblastic tumor: endoscopic and magnetic resonance imaging findings. Case Rep Oncol.

[3] Liu W, Zhou J, Yang J, Huang X (2022). A multicenter retrospective study of epithelioid trophoblastic tumors to identify the outcomes, prognostic factors, and therapeutic strategies. Front Oncol.

[4] Shaaban AM, Rezvani M, Haroun RR (2017). Gestational trophoblastic disease: clinical and imaging features. Radiographics.

[5] Ngan HY, Seckl MJ, Berkowitz RS (2018). Update on the diagnosis and management of gestational trophoblastic disease. Int J Gynaecol Obstet.

[6] Horowitz NS, Goldstein DP, Berkowitz RS (2017). Placental site trophoblastic tumors and epithelioid trophoblastic tumors: biology, natural history, and treatment modalities. Gynecol Oncol.

[7] Li J, Du Z, Xu T, Li C, Ba S, Zhu H (2024). Epithelioid trophoblastic tumor with lung metastasis: a case report and literature review. Medicine (Baltimore).

[8] McDonald K (2026). Gestational trophoblastic disease tumours and mimics: an up-to-date review of morphology and immunocytochemistry to aid the diagnosis. Diagn Histopathol.

[9] Stichelbout M, Devisme L, Franquet-Ansart H, Massardier J, Vinatier D, Renaud F, Kerdraon O (2016). SALL4 expression in gestational trophoblastic tumors: a useful tool to distinguish choriocarcinoma from placental site trophoblastic tumor and epithelioid trophoblastic tumor. Hum Pathol.

[10] Basto R, Brandão Rêgo I, Correia Magalhães J (2021). Epithelioid trophoblastic tumour presenting with scalp lesions. Eur J Case Rep Intern Med.

[11] Kageyama S, Kanoto M, Sugai Y, Suto T, Nagase S, Osakabe M, Hosoya T (2016). MR imaging of uterine epithelioid trophoblastic tumor: a case report. Magn Reson Med Sci.

[12] Aniţei MG, Lazăr DE, Pleşca RA, Terinte C, Iulian R, Viorel S (2021). Uterine epithelioid trophoblastic tumor in a 44-year-old woman: a diagnostic dilemma. Clin Pract.

[13] Pisani D, Calleja-Agius J, Di Fiore R (2021). Epithelioid trophoblastic tumour: a case with genetic linkage to a child born over seventeen years prior, successfully treated with surgery and pembrolizumab. Curr Oncol.

[14] Shih IM, Kurman RJ (2004). p63 expression is useful in the distinction of epithelioid trophoblastic and placental site trophoblastic tumors by profiling trophoblastic subpopulations. Am J Surg Pathol.

[15] Trecourt A, Donzel M, Gaillot-Durand L (2025). SALL4 as a useful marker for the distinction of various gestational trophoblastic disease subtypes: choriocarcinoma from other trophoblastic lesions and early complete hydatidiform mole from partial mole and Nonmolar villi. Am J Surg Pathol.

[16] Frijstein MM, Lok CA, van Trommel NE (2019). Management and prognostic factors of epithelioid trophoblastic tumors: results from the International Society for the Study of Trophoblastic Diseases database. Gynecol Oncol.

[17] Tempfer C, Horn LC, Ackermann S (2016). Gestational and non-gestational trophoblastic disease: guideline of the DGGG, OEGGG and SGGG (S2k level, AWMF registry no. 032/049, December 2015). Geburtshilfe Frauenheilkd.

[18] Ngan HY, Seckl MJ, Berkowitz RS (2021). Diagnosis and management of gestational trophoblastic disease: 2021 update. Int J Gynaecol Obstet.

[19] Soper JT (2021). Gestational trophoblastic disease: current evaluation and management. Obstet Gynecol.

[20] Powles T, Savage P, Short D, Young A, Pappin C, Seckl MJ (2006). Residual lung lesions after completion of chemotherapy for gestational trophoblastic neoplasia: should we operate?. Br J Cancer.

